# Splenic metastases – hematogenous disease or invasive peritoneal implants? Case reports of two patients

**DOI:** 10.1016/j.ijscr.2020.05.086

**Published:** 2020-06-11

**Authors:** Paul H. Sugarbaker, Ebony R. Hoskins

**Affiliations:** a Center for Gastrointestinal Malignancies, MedStar Washington Hospital Center, Washington, DC, USA; b Section on Gynecologic Oncology, MedStar Washington Hospital Center, Washington, DC, USA

**Keywords:** Peritoneal metastases, Hematogenous metastases, Appendiceal mucinous neoplasm, Pseudomyxoma peritonei, Gastrointestinal cancer, Ovarian cancer, Cytoreductive surgery, Debulking surgery, HIPEC, Intraperitoneal chemotherapy, Case report

## Abstract

**Background:**

Metastases from gastrointestinal and gynecologic malignancy may occur through lymphatic channels, through venules of the cancer or into the peritoneal space. The metastatic process involves lymphatic, hematogenous or transcoelomic dissemination.

**Methods:**

Two patients with splenic metastases are presented. Their clinical course is presented in an attempt to identify the route of cancer dissemination to the spleen. Lymphatic, hematogenous, or transcoelomic patterns of cancer dissemination are possible. Transcoelomic cancer dissemination into the peritoneal space results in peritoneal metastases.

**Results:**

In a woman with ovarian cancer peritoneal metastases, a large lesion within the parenchyma of the spleen was described. This disease was present at multiple other sites as peritoneal metastases but no hematogenous or lymphatic metastases. A second patient with mucinous appendiceal neoplasm with peritoneal metastases was studied. Hematogenous metastases from this malignancy is extremely rare and lymphatic metastases were not present at the time of right colon resection. After studying these two patients, our hypothesis is that splenic metastases result from transcoelomic dissemination to the splenic hilum or splenic notches with progression of disease into the parenchyma of the spleen.

**Conclusions:**

When oncologists are considering aggressive local-regional treatments for peritoneal metastases important patient management decisions are influenced by the presence versus absence of hematogenous metastases. We suggest that the presence of splenic metastases does not indicate systemic (hematogenous) or lymphatic metastatic process but an extension of peritoneal metastases at the hilum of the spleen or within splenic notches deep into the parenchyma of the spleen.

## Highlights

• Determine metastases as hematogenous, lymphatic or transcoelomic.
• In the past splenic metastases was assumed to be hematogenous.
• Splenic metastases may occur in the absence of hematogenous dissemination.
• Gastrointestinal or gynecologic splenic metastases are likely implants.

## 1 Introduction

When cancer cells gain access to the bloodstream, it is possible that they can stick to the vessel walls of an organ, implant by means of neovascularity and progress to a nodule that is visible to the eye and detectable radiologically. This complex series of events occurs more frequently in some organs than in others. Organs such as the liver have portal vascular systems with a reduced velocity of blood flow [1]. Also, the adrenal gland and bone marrow has a low velocity of venous blood flow and high incidence of metastases [2]. At rest, the capillaries of the lungs filter the complete blood flow of cancer cells every minute and so have a high incidence of hematogenous metastases [3]. This may help explain the high incidence of hematogenous metastases in these organs. One organ that has a high blood flow but almost never develops hematogenous metastases is the spleen [4,5]. It has a generous arterial blood flow to bring in circulating cancer cells but it is hypothesized that the large accumulation of immune cells almost always prevents the development of splenic metastases. It has been suggested that patients who have a large burden of hematogenous metastases may develop splenic metastases from cells entering the spleen through the splenic artery.

A second possible route for cancer cells to move from gastrointestinal or ovarian primary cancer to the spleen is the lymphatic system. However, no lymphatic channels from the gut or ovaries to the spleen exist. It is difficult, even impossible, to suggest that the cancer cells that progress to splenic metastases reach the spleen through the lymphatics.

A third route for cancer cells to follow to develop distant disease is through a body cavity. This type of spread occurs on pleural, pericardial or peritoneal surfaces. Peritoneal metastases frequently occur from gastrointestinal and gynecologic malignancy. The same sequence of cancer cell adherence, vascularization, and progression occurs with peritoneal metastases as with hematogenous metastases.

In this case report two patients with extensive peritoneal metastases also developed splenic metastases. The clinical, radiologic and histopathologic characteristics of these two patients are studied in an attempt to determine if the route of cancer dissemination to splenic parenchyma is hematogenous or transcoelomic.

## 2 Materials and methods

Cytoreductive surgery which requires peritonectomy procedures and visceral resections was performed on our 2 patients as previously described [6]. As the abdomen is explored the peritoneal cancer index (PCI) was determined to provide an objective assessment of the extent of disease [7]. This assessment determines the size of peritoneal nodules in 13 abdomino-pelvic regions with the abdomen open at the time of cytoreductive surgery. The score ranges from 0 (no visible disease) to 39 (tumor nodules greater than 5 cm in all 13 abdomino-pelvic regions). To assess the extent of cancer resection by cytoreductive surgery, the completeness of cytoreduction (CC) score was used. This assessment measures the largest nodule or layer of cancer in 13 abdomino-pelvic regions that remains after the best efforts at cytoreductive surgery. No visible disease was scored as CC-0. CC-1 indicated all nodules or layers are less than 0.25 cm, CC-2 nodules from 0.25 to 2.5 cm and CC-3 greater than 2.5 cm [7]. At the completion of a complete cytoreduction in order to treat microscopic residual disease, hyperthermic intraperitoneal chemotherapy (HIPEC) was used. Mitomycin C or cisplatin in a large volume of 1.5% dextrose peritoneal dialysis solution at 42 °C is used to treat all abdominal and pelvic surfaces [8]. For the initial 5 days following surgery, 5-fluorouracil or paclitaxel is instilled as early postoperative intraperitoneal chemotherapy [8].

Data on these patients were prospectively recorded and then retrospectively reviewed at an academic institution. This research work has been reported in line with the SCARE criteria [9]. This study was registered as a case report on the www.researchregistry.com website with UIN 5552.

## 3 Case report

### 3.1 Advanced ovarian cancer with splenic metastases

A 59-year-old gravida 3, para 3 woman was referred to the gynecologic oncology service because of a pelvic mass.

**Fig. 1:**
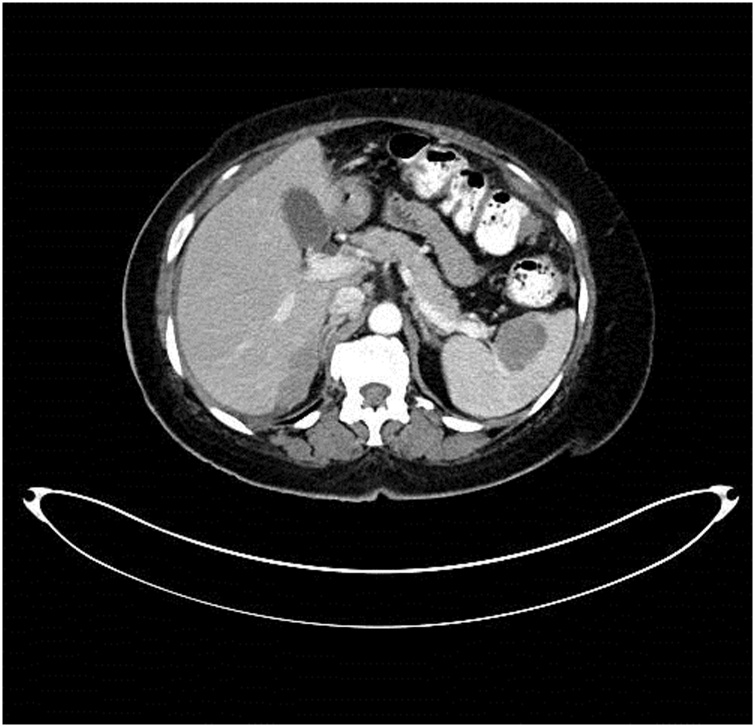
This 59-year-old woman presented with abdominal pain and an enlarging right ovary. Peritoneal metastases were present beneath the right hemidiaphragm and within the pelvis. A 3.8 cm mass was located within the splenic parenchyma and identified by the radiologist as “splenic metastases”.
▪ Biopsy of the abdominal wall mass revealed adenocarcinoma likely of gynecologic origin.
▪ Because of the large extent of disease and the presumed hematogenous metastases to the spleen, neoadjuvant chemotherapy with carboplatin and paclitaxel was recommended. Four cycles were planned. Pretreatment CA125 tumor marker was 1283 units/mL.
▪ In January 2020, preoperative evaluation revealed significant improvement of abdominal discomfort. CA-125 had decreased to 39.1 units/mL. Repeat CT scan showed reduction of tumor volume at all sites but the disease had persisted. The splenic metastasis had responded to the systemic chemotherapy but was still visible by CT (Fig. 2).

▪ In March 2019, the patient complained of abdominal pain. Pelvic sonogram showed a 6.0 × 4.6 × 3.6 cm left ovarian cyst and 4.9 × 3.6 × 3.9 cm complex cystic mass enlarging the right ovary. After completing some personal business out of the USA, a radiologic study was performed.
▪ In August 2019, CT of abdomen and pelvis showed soft tissue nodules up to 5 cm in diameter compatible with peritoneal metastases beneath the right hemidiaphragm. A 3.8 cm mass was completely intrasplenic (Fig. 1). A lobular mass filled the pelvis and was inseparable from the rectosigmoid colon. It measured 15 × 11 × 8 cm and caused a mass effect on the urinary bladder. An anterior abdominal wall mass was thought to occur within an umbilical hernia. CT showed no evidence of pleural tumor, retroperitoneal lymph nodal disease or liver metastases.

**Fig. 2:**
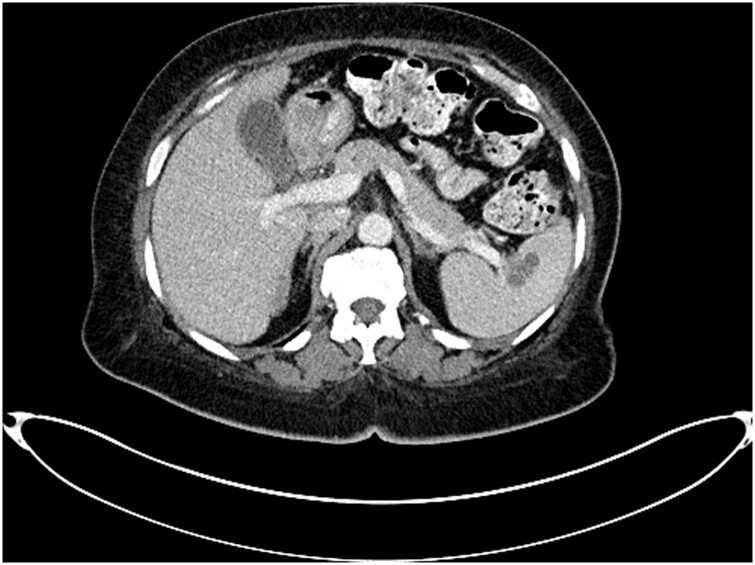
The 59-year-old woman with ovarian cancer stage IIIc was given 4 cycles of carboplatin and paclitaxel. Her CA-125 decreased from 1283 to 39.1. The splenic metastasis responded to systemic chemotherapy but was still visible.
▪ On January 13, 2020 at the abdominal exploration, the PCI was estimated to be 23. A debulking procedure was performed. Resections included tumor from the anterior abdominal wall, uterus with bilateral salpingo-oophorectomy, tumor masses anterior to the rectosigmoid colon, appendectomy and greater omentectomy. Tumor beneath the hemidiaphragms and the spleen were not removed. The resection was scored as a CC-3 cytoreduction.
▪ On January 17, 2020, the patient was discharged from the hospital without adverse events. Plan is additional carboplatin and paclitaxel chemotherapy.

### 3.2 Appendiceal mucinous neoplasm with splenic metastases

**Fig. 3:**
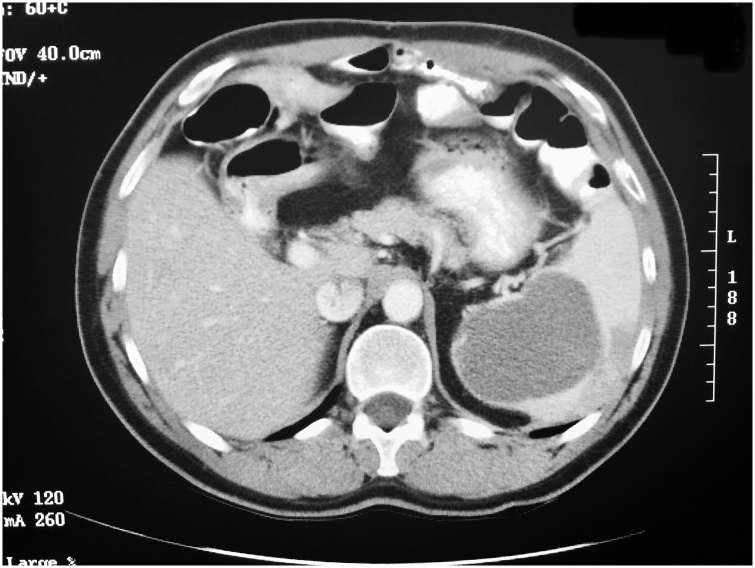
A 48-year-old man developed right lower quadrant pain and was taken to the operating theater. A ruptured appendix was associated with widespread mucinous tumor within the peritoneal space. He was treated with 5-fluorouracil plus leucovorin chemotherapy. Seven years after the appendectomy a repeat CT scan shows a 7 cm in diameter cystic mass within the central portion of the spleen.
▪ On November 24, 2004, exploratory laparotomy was performed. The mucinous adenocarcinoma was contained within the splenic capsule. The spleen with a portion of the left hemidiaphragm was removed intact. No HIPEC or EPIC was used.
▪ On March 8, 2013, the patient died but cause of death was unknown.

▪ On June 1997, a 48-year-old male complained of right lower quadrant pain and fever. On an emergency basis he was taken to the operating theater where he was found to have a ruptured appendix. Not only was extensive inflammation present but there was mucin widely disseminated within both abdomen and pelvis. A right colon resection with anastomosis was performed. Pathology report showed a mucinous adenocarcinoma of the appendix. Four lymph nodes were negative for neoplasm. After recovery from surgery he underwent chemotherapy with 5-fluorouracil and leucovorin. When disease progression occurred, irinotecan was used as systemic chemotherapy.
▪ On May 9, 2000, a repeat CT scan showed tumor masses beneath the right hemidiaphragm. This was confirmed by biopsy of a perihepatic mass. Tumor masses were seen within the porta hepatis, within the omental bursa and greater omentum. A mass 7 cm in diameter is present within the old ileocolic anastomosis and causing partial small bowel obstruction. The left upper quadrant of the abdomen and spleen showed no evidence of mucinous adenocarcinoma. CA 19-9 tumor marker had increased to 527.8.
▪ On November 28, 2000, an 8 -h cytoreductive surgery was performed. At exploration the PCI was 17. Extensive peritonectomy of the right upper quadrant and pelvis were performed along with greater and lesser omentectomy. HIPEC with mitomycin C and EPIC with 5-fluorouracil were performed in the operating theater and for the first 5 postoperative days. The patient was discharged without adverse events.
▪ In June 2004, CT performed on routine follow-up showed a mass enlarging within the spleen. CA 19-9 had reached 2010 on November 23, 2004. CEA was 9.
▪ On November 23, 2004, repeat CT showed a 7 cm in diameter cystic mass within the central part of the spleen (Fig. 3). No other sites of disease were noted on CT.

## 4 Discussion

### 4.1 Classification of splenic metastases reported in the oncology literature

Although splenic metastases are uncommon, there are numerous reports in the oncology literature of their occurrence. If splenic metastases have a hematogenous route of dissemination they should be reported for cancers that have their primary site outside of the abdomen and pelvis. They should occur, at least in some patients, as multiple sites of disease within the splenic parenchyma. They should be associated with other sites of hematogenous metastases such as lung, liver and bone marrow. Finally, they should occur in the absence of peritoneal metastases. Neither of our two patients had these associated clinical features for a hematogenous origin of splenic metastases.

If splenic metastases have a transcoelomic route, the primary site should be limited to the abdomen and pelvis (gastrointestinal and gynecologic malignancy). They should occur as a single intraparenchymal splenic mass or only a few masses because access of cancer cells through the splenic capsule does not frequently occur. Also, since the deepest crypts in which cancer cells may become entrapped are at the hilum, a preponderance of the splenic metastases would be near the hilum of the spleen. The splenic masses from invasive implants should not be associated with systemic metastases to liver, bone marrow or lung. They should be associated with other sites of peritoneal metastases. An exception to this criteria would be patients, as our patient 2, who had cytoreductive surgery and HIPEC and EPIC to eradicate peritoneal metastases at a prior time. In the ovarian cancer patient and mucinous adenocarcinoma of the appendix patient presented, transcoelomic peritoneal metastases would be selected as the route for cancer cells to reach the splenic parenchyma.

### 4.2 Clinical relevance of the causation of splenic metastases

It is important to establish if metastases are disseminated by a hematogenous route or if they are transcoelomic peritoneal metastases. Judgments regarding the extent of a cytoreductive surgery often depend on the surgeon’s confidence that the tumor masses being removed by cytoreductive surgery are limited to the peritoneal surface rather than of systemic disease. A local-regional treatment such as cytoreductive surgery is unlikely to be of long-term benefit with hematogenous dissemination of disease. If splenic metastases are from a hematogenous source, the resection of the spleen during cytoreductive surgery would not be indicated. However, if splenic metastases are a manifestation of splenic invasion by peritoneal metastases, inclusion of the spleen and the disease within the organs resected would be completely reasonable. This is why it is important to establish the origin of the splenic metastases in performing surgery for peritoneal metastases, especially ovarian malignancy.

### 4.3 Splenic metastases may occur in neoplasms that do not produce hematogenous metastases

Hematogenous metastases do occur in patients with ovarian cancer but were not seen by CT in the patient reported here. However, a hematogenous route for splenic metastases with ovarian cancer may be possible. However, splenic metastases have been reported frequently with pseudomyxoma peritonei of either ovarian or appendiceal origin [10]. Pseudomyxoma peritonei is a disease process that disseminates itself within the peritoneal cavity in a characteristic pattern of distribution [11]. However, in contrast to ovarian cancer, hematogenous metastases with this disease are extremely rare and perhaps do not occur. The presence of splenic metastases in patients with pseudomyxoma peritonei argues strongly in favor of this pattern of metastases occurring as a result of transcoelomic movement of cancer cells rather than hematogenous cancer dissemination.

### 4.4 Splenic metastases occurring in the absence of hematogenous metastases at other sites

Another reason to classify splenic metastases in serous ovarian cancer as transcoelomic rather than hematogenous concerns the mechanism for metastatic disease to the spleen. Having cancer cells enter the spleen from the systemic blood circulation through the splenic artery could occur. However, if it did occur, one would expect metastases also within the parenchyma of the liver and at other systemic sites. In the patient reported here, the only site identified by CT for possible hematogenous dissemination of serous ovarian cancer was the spleen. All of this patient’s disease at other sites was limited to peritoneal surfaces. More than likely these splenic metastases were from a peritoneal spread of the disease that becomes invasive into the splenic parenchyma.

### 4.5 Access of intraperitoneal cancer cells to the spleen

If the splenic metastases are in reality an invasion of the parenchyma of the spleen by peritoneal implants, how do the cancer cells gain access? One hypothesis is that the cancer cells enter the splenic parenchyma, either at the hilum or through the splenic notches. In fetal life, the spleen is a multilobular structure. These lobules coalesce in fetal life to form in the human a single structure. However, frequently the fusion of the splenic lobules is not perfect and the “splenic notches” will persist [12]. Cancer cells trapped within crypts at the splenic hilum or surface notches may progress into the soft splenic parenchyma following the path of least resistance beneath the capsule of the spleen as the cancer cells proliferate and the tumor mass enlarges.

In summary, we postulate two groups of patients who develop splenic metastases. One group has a large burden of hematogenous metastases to liver, lung and bone marrow. High levels of circulating cancer cells may be expected to enter the splenic parenchyma via the splenic artery and progress to tumor masses demonstrated by CT. These patients are not candidates for splenectomy.

The second group of patients, like our two cases, have peritoneal metastases and no evidence by CT of hematogenous cancer dissemination. Splenic tumor masses within the spleen are accompanied by other sites of transcoelomic cancer dissemination. These patients should be considered for splenectomy as part of an attempt to achieve complete cytoreductive surgery. In this regard, the concept of splenic metastases as an unusual manifestation of peritoneal metastases has important implications in the optimal surgical management of gastrointestinal and gynecologic cancers with peritoneal dissemination.

## Declaration of Competing Interest

Paul H. Sugarbaker has no conflicts of interest to declare.

Ebony R. Hoskins has no conflicts of interest to declare.

## Sources of funding

Data management and secretarial support provided by Foundation for Applied Research in Gastrointestinal Oncology.

## Ethical approval

Local IRB-approval for this case report was not required:

MedStar Health Institutional Review Board has determined that a case report of less than three [3] patients does not meet the DHHS definition of research (45 CFR 46.102(d)(pre-2018)/45 CFR 46.102(l)(1/19/2017)) or the FDA definition of clinical investigation (21 CFR 46.102(c)) and therefore are not subject to IRB review requirements and do not require IRB approval.

This case report is of 2 patients.

## Consent

Informed consent was requested with one patient and through surviving family members of another for publication of this case report.

## Author contribution

Paul H. Sugarbaker, MD: study concept or design, data collection, data analysis or interpretation, writing the paper.

Ebony R. Hoskins, MD: study concept or design, data collection, data analysis or interpretation.

## Registration of research studies

This study was registered as a case report on the www.researchregistry.com website with UIN 5552.

## Guarantor

Paul H. Sugarbaker, MD.

## Provenance and peer review

Not commissioned, externally peer-reviewed.
